# Mandatory Food Fortification Vehicles in Indonesia: Consumption Patterns and Demand Elasticities from Susenas 2024

**DOI:** 10.3390/foods15183253

**Published:** 2026-09-15

**Authors:** Drajat Martianto, Azis Boing Sitanggang, Jonathan Louis Gorstein, Nina Sardjunani, Roza Kartika, Elsa Fajriah, Milatul Mustaqimah

**Affiliations:** 1 Division of Food and Nutrition Policy, Faculty of Medicine and Nutrition, IPB University, Bogor 16680, Indonesia; 2 Division of Food Science and Technology, Faculty of Engineering and Technology, IPB University, Bogor 16680, Indonesia; 3 Graduate School of Agricultural Science, Tohoku University, Sendai 980-8572, Miyagi, Japan; 4 Gates Foundation, Seattle, WA 98109, USA; 5 Indonesian Nutrition Foundation for Food Fortification (KFI), Komplek Bappenas A1, Jl. Siaga Raya, Pejaten Barat, Jakarta Selatan 12510, Indonesia

**Keywords:** food fortification, demand elasticity, consumption patterns, demand elasticity, Almost Ideal Demand System, palm cooking oil, wheat flour, salt, Indonesia, Susenas

## Abstract

Micronutrient deficiencies remain a major public health challenge in Indonesia, and mandatory food fortification has been implemented to improve population micronutrient intake. However, the potential reach of fortification vehicles depends not only on regulatory compliance but also on household consumption patterns and demand responsiveness. This study analyzed consumption patterns and estimated the price and expenditure elasticities of mandatory fortification vehicles—wheat flour and its derivatives, palm cooking oil, and salt—using data from the March 2024 National Socioeconomic Survey (Susenas), covering 343,479 households. Demand elasticities were estimated using the Almost Ideal Demand System (AIDS), with prices proxied by unit values adjusted for quality effects. All three commodities were widely consumed, with national participation rates of 99.3% for wheat flour and its derivatives, 96.2% for palm cooking oil, and 97.5% for salt. The national own-price elasticities were −0.848 for wheat flour and its derivatives, −0.499 for palm cooking oil, and −0.844 for salt, indicating generally price-inelastic demand. However, demand responsiveness varied across expenditure groups and urban–rural settings. Wheat flour showed greater price sensitivity among lower-expenditure households, while palm cooking oil and salt exhibited more heterogeneous patterns. Expenditure elasticities were positive for all three commodities, although their magnitude varied across population groups. These findings indicate that fortification vehicles differ in consumption distribution and demand responsiveness and that participation rates should be interpreted as potential reach rather than actual coverage of fortified products. Consideration of consumption patterns, affordability, and demand responsiveness can inform a complementary multi-vehicle approach to food fortification in Indonesia.

## 1. Introduction

Micronutrient deficiencies remain a major global public health challenge, affecting more than two billion people, particularly in low- and middle-income countries [1,2]. Deficiencies in essential nutrients such as iron, zinc, iodine, and vitamin A are associated with increased morbidity, reduced productivity, and impaired cognitive development, especially among women and children [3]. One of the most effective and sustainable strategies to address these deficiencies is large-scale food fortification (LSFF), which involves adding essential micronutrients to widely consumed staple foods [2]. Evidence from systematic reviews shows that LSFF can significantly reduce the prevalence of anemia, iodine deficiency disorders, and neural tube defects while improving overall micronutrient status in vulnerable populations [1,4]. In addition, fortification is widely recognized as a highly cost-effective intervention, as most programs demonstrate favorable cost-effectiveness ratios relative to gross domestic product (GDP) per capita thresholds [5]. Despite its proven effectiveness and widespread implementation in more than 140 countries, ensuring adequate coverage and equitable distribution of benefits remains challenging. Variations in consumption patterns of fortification vehicles, inconsistent compliance with fortification standards, and limited monitoring systems often constrain program effectiveness, particularly among low-income and rural populations [6]. These challenges highlight that the success of fortification programs depends not only on regulatory frameworks and production capacity but also on the extent to which fortification vehicles are consumed across different population groups.

Micronutrient deficiencies continue to pose a significant public health concern globally and in Indonesia. Anemia remains one of the most prevalent micronutrient-related conditions, affecting 30.7% of women aged 15–49 years and 35.5% of pregnant women globally in 2023, while 39.8% of children aged 6–59 months were estimated to have anemia in 2019 [7]. In Indonesia, the 2023 National Health Survey reported anemia among 27.7% of pregnant women and 23.8% of children under five years, while vitamin A deficiency affected 9.8% of children [8]. These persistent micronutrient deficiencies underscore the need for population-level strategies to improve micronutrient intake. In response, Indonesia has implemented mandatory fortification policies for widely consumed food vehicles, including iodized salt, iron-fortified wheat flour, and vitamin A-fortified cooking oil, while the National Medium-Term Development Plan 2025–2029 promotes rice fortification [9,10]. Because the nutritional benefits of fortification depend on whether fortified vehicles are actually consumed, understanding household consumption of these vehicles is essential for assessing the potential reach and equity of fortification policies. While these policies provide a strong regulatory foundation, their effectiveness ultimately depends on household consumption behavior and the accessibility of fortification food vehicles across socioeconomic and geographic groups. However, consumption alone does not fully characterize the economic conditions under which households access these food vehicles. Household expenditure patterns and demand responsiveness to prices provide complementary information on affordability and the potential sensitivity of consumption to economic changes. Price elasticity indicates how strongly consumption may respond to changes in prices, while expenditure elasticity indicates how consumption varies with household resources. These measures are therefore relevant for understanding the economic accessibility and resilience of different fortification vehicles across population groups [11].

However, empirical evidence linking mandatory fortification policies with household consumption dynamics remains limited. Existing studies have largely focused on regulatory compliance, production systems, or nutritional outcomes, with little attention to demand behavior. The present study focuses on iodized salt, iron-fortified wheat flour, and vitamin A-fortified cooking oil because these commodities represent widely consumed food vehicles subject to mandatory fortification policies in Indonesia and provide important delivery channels for different micronutrients [12]. Importantly, mandatory fortification does not imply the same level of population exposure across food vehicles. A fortification vehicle does not provide the same population exposure simply because it is subject to mandatory fortification; its potential population reach depends on how widely and consistently the commodity is consumed across population groups [6]. Because wheat flour, palm cooking oil, and salt differ in their dietary roles, consumption patterns, and market characteristics, their potential reach may therefore differ across socioeconomic and residential groups. These differences may also result in different responses to changes in prices and household expenditure.

In particular, no nationally representative study has examined how household demand for mandatory fortification vehicles responds to price and expenditure changes or how these patterns differ across expenditure groups and urban–rural areas. Global studies suggest that fortification coverage may vary significantly between poor and non-poor households and between urban and rural populations [4]. However, without disaggregated estimates of demand elasticities, it remains difficult to assess whether complementary policies are needed to improve equity and access. Place of residence is an important dimension of equity because differences in market access, food availability, prices, and dietary patterns may influence both consumption and demand responsiveness [13,14]. Understanding these differences is therefore important for assessing whether the potential benefits of fortification reach population groups equitably.

To quantify these demand responses, the Almost Ideal Demand System (AIDS) model developed by Deaton and Muellbauer (1980) [11] provides a theoretically consistent framework for estimating price and expenditure elasticities. However, its application to food fortification and demand analysis in Indonesia remains limited. This is the first study using Susenas 2024 to estimate demand elasticities for mandatory fortification vehicles in Indonesia.

Based on demand theory and the existing evidence, we hypothesized that (1) consumption patterns and potential population reach would differ across mandatory fortification vehicles; (2) demand for these vehicles would generally be price inelastic, but price responsiveness would be greater among lower-expenditure households; and (3) demand responses would vary across expenditure groups and between urban and rural households.

To address this gap, the present study provides the first comprehensive national analysis that simultaneously examines household consumption patterns and demand elasticities of mandatory fortification vehicles in Indonesia using the 2024 National Socioeconomic Survey (Susenas) and the Almost Ideal Demand System (AIDS). Specifically, this study (1) analyzes household consumption patterns of key fortification vehicles, including wheat flour, cooking oil, and salt; (2) estimates price and expenditure elasticities; and (3) evaluates differences across expenditure quintiles and areas of residence (urban and rural). By combining economic demand analysis with nutrition policy considerations, this study provides an evidence base to inform the design and strengthening of food fortification policies in Indonesia.

## 2. Materials and Methods

### 2.1. Study Design

This study employed a quantitative, cross-sectional design based on secondary analysis of household-level data from the March 2024 National Socioeconomic Survey (Susenas). The analysis focused on household consumption patterns and demand responsiveness for three mandatory food fortification vehicles in Indonesia: wheat flour and its derivatives, palm cooking oil, and salt. Demand responsiveness was assessed using price and expenditure elasticities, with particular attention to differences across expenditure quintiles and urban–rural residence.

The analysis applies the Almost Ideal Demand System (AIDS) model developed by Deaton and Muellbauer (1980) [11]—a widely used econometric framework that allows simultaneous estimation of price and expenditure elasticities while maintaining consistency with microeconomic demand theory. The AIDS model is particularly suitable for cross-sectional household data and has been extensively applied in empirical food demand analyses in developing countries [11,15,16].

### 2.2. Data Source and Availability

This study is a secondary analysis of cleaned household-level microdata from the March 2024 National Socioeconomic Survey (Susenas), conducted by Statistics Indonesia (BPS). The analytical dataset contained 343,479 households across 38 provinces and 514 districts/cities in Indonesia. Susenas collects detailed socioeconomic and consumption information through structured face-to-face interviews with household respondents [17]. The survey uses standardized questionnaires (Table S1) administered by Statistics Indonesia (BPS), including the Core Questionnaire (KOR/VSEN24.K), which provides demographic and socioeconomic characteristics, and the Consumption and Expenditure Questionnaire (KP/VSEN24.KP), which contains detailed information on household food and non-food consumption, including quantities and expenditures. BPS implements standardized survey manuals, enumerator training, supervision, and data quality and completeness procedures as part of the survey’s quality assurance process. No additional household-level exclusion criteria were applied in this secondary analysis beyond these procedures described in Section 2.3. Food consumption data were collected using a seven-day recall method, from which unit values were calculated as proxies for prices based on the ratio of expenditure to quantity. While widely used in demand analysis, unit values may reflect both price and quality variations and should therefore be interpreted with caution [18]. Susenas data are publicly accessible through Statistics Indonesia (https://silastik.bps.go.id, accessed on 5 June 2026), subject to standard data access procedures; at the time of submission, no accession number is assigned, but access details can be provided upon request to facilitate replication. Sampling weights were applied throughout the descriptive estimation and data aggregation procedures to obtain population-representative estimates. For the demand-system analysis, household-level observations were aggregated into weighted percentile groups before estimation.

### 2.3. Data Processing and Commodity Classification

Households were the primary unit of observation for data processing and construction of consumption, expenditure, and participation measures. For the demand-system estimation, household-level observations were subsequently aggregated into weighted percentile groups defined by urban–rural residence and expenditure quintile. All data processing and statistical analyses were conducted using Stata 17 (StataCorp LLC, College Station, TX, USA) and IBM SPSS Statistics 25 (IBM Corp., Armonk, NY, USA). The raw data, originally provided in .dbf format along with data dictionaries and commodity codes, were first converted into .dta and .sav formats while preserving metadata and coding structures. The core (KOR) and consumption (KP) datasets were subsequently merged using a unique household identifier.

Data cleaning procedures were performed to address missing values, inconsistencies, and outliers. A data cleaning procedure was applied to address extreme observations and ensure data validity. Rather than relying solely on conventional statistical trimming (e.g., ±3 standard deviations), this study adopts a region-based approach using median values at the district (kabupaten/kota) level combined with an urban–rural classification to detect and correct implausible expenditure and quantity values. Missing or outlier unit values were imputed using median values at the lowest available geographic level to minimize measurement bias.

For the demand-system analysis, food consumption items were aggregated into 12 mutually exclusive commodity groups: rice, wheat flour and derivatives (including noodles, bread, and biscuits converted into wheat flour equivalents), other staple foods, fish, meat, eggs and dairy products, vegetables and fruits, palm cooking oil, other oils and fats, legumes, salt, and a residual “other food” category. Conversion factors were applied to processed wheat-based foods to express consumption in wheat flour equivalents, ensuring comparability across food items [19]. The residual category captures all remaining food expenditures and allows the demand system to satisfy the adding-up condition [11].

Variables were constructed by expressing consumption as weekly per capita expenditure (IDR/capita/week), while prices were proxied using unit values and total food expenditure was calculated as the aggregate of all food expenditures [20]. The dataset was further disaggregated by expenditure quintiles (Q1–Q5) and area of residence (urban and rural) to enable subgroup analysis. These procedures follow standard practices in household demand analysis to ensure consistency and comparability of estimates. Budget shares were defined as the ratio of commodity expenditure to total food expenditure, and total food expenditure was calculated as the sum of all food-related expenditures. All descriptive statistics and aggregation procedures were performed using sampling weights (weind) to maintain national representativeness.

Several assumptions and limitations should be considered when interpreting the results. First, household food expenditures were assumed to approximate actual consumption, excluding food waste and changes in food stocks. Because Susenas records food consumption at the household level, per-capita estimates assume equal intra-household distribution and may not fully reflect differences in age-, sex-, and physiological requirement-specific consumption patterns. This limitation is particularly relevant when interpreting potential nutrient exposure rather than commodity consumption alone [21,22]. Second, the treatment of foods consumed as ingredients in processed foods differs across commodities. For wheat flour, direct purchases and wheat-based processed foods consumed at home and outside the home were converted into wheat flour equivalents using standardized conversion factors. However, the contribution of salt and palm cooking oil contained in processed foods and foods consumed away from home could not be fully identified from the available data. Thus, consumption estimates for these commodities may underestimate total exposure. Third, prices were proxied using unit values, which were adjusted for observable differences associated with household characteristics [18]. In addition, the analysis uses household expenditure as a proxy for welfare and does not distinguish between fortified and non-fortified products. Therefore, the results characterize household consumption, demand responsiveness, affordability, and potential reach rather than actual fortification coverage or nutrient intake. These limitations are inherent to the use of household consumption and expenditure survey data and should be considered when translating the findings into fortification policy [21].

### 2.4. Price Construction and Quality Adjustment

Prices were proxied using unit values, defined as the ratio of household expenditure to quantity consumed for each commodity. To address potential quality effects inherent in unit values, a quality adjustment procedure following Deaton (1988) [18] was implemented. Specifically, the logarithm of unit values was regressed on household characteristics, including the age of the household head, urban–rural residence, total expenditure (log-transformed), and the gender of the household head, using only observations with positive consumption. The predicted component from this regression was interpreted as quality-related variation and subtracted from the observed unit values to obtain quality-adjusted prices.

To account for spatial price variations, median unit values were calculated at the district and urban–rural levels and used as reference prices. For households with zero consumption, prices were imputed using these median values adjusted by the estimated quality component. Missing predicted prices were imputed using sample means to ensure dataset completeness. This approach follows standard practices in demand analysis to separate price variation from quality heterogeneity and measurement error [18].

### 2.5. Treatment of Zero Consumption and Data Aggregation

Zero consumption was addressed using a two-step procedure. First, probit models were estimated at the household level for each commodity to model the probability of consumption participation. Based on these estimates, the Inverse Mills Ratio (IMR) was computed for each observation [23]. The IMR was then included as an additional regressor in the AIDS demand system to correct for potential selection bias arising from households that did not consume certain commodities during the survey period [23,24].

Following household-level estimation, data reduction was performed to improve estimation efficiency, given the large sample size of the 2024 Susenas (343,479 households). Households were first classified into national expenditure quintiles and subsequently divided into 100 percentile groups within each combination of urban–rural classification and expenditure quintile. The dataset was then collapsed by these groups, where key variables were averaged using sampling weights to construct grouped units of analysis that retained the representative characteristics of the underlying population. Real expenditure was constructed using the log-linear Stone price index, which is defined as a weighted average of logarithmic prices with budget shares as weights. Specifically, the index was calculated after data aggregation using predicted prices and excluding the residual “other” commodity category. Real expenditure was then obtained by deflating total food expenditure with this index [11].

### 2.6. Model Specification and Analytical Approach

To formally estimate household demand behavior, the econometric specification of the model is presented below. The demand system is estimated using the Almost Ideal Demand System (AIDS), in which the budget share equation is defined as shown in Equation (1) [11]:

(1) $$w_{i}=\alpha_{i}+\sum_{j=1}^{n}\gamma_{ij}\ln p_{j}^{*}+\beta_{i}\ln\left(X/P^{*}\right)+\varepsilon_{i}$$

where $w_{i}$ represents the budget share of commodity *i*, $p_{j}^{*}\;$denotes quality-adjusted prices, X is total food expenditure, P* is the Stone price index, and $\varepsilon_{i}\;$is an error term.

Equation (2) defines the Stone price index.

(2) $$\ln P^{*}=\sum_{k}w_{k}\ln p_{k}^{*}$$

where P* denotes the Stone price index constructed using the budget shares and quality-adjusted prices. In the empirical implementation, the index is calculated using predicted prices and excludes the residual “other” commodity category.

To ensure consistency with microeconomic demand theory, the following restrictions are imposed. The adding-up restriction (Equation (3)):

(3) $$\sum_{i}\alpha_{i}=1,\sum_{i}\beta_{i}=0,\sum_{i}\gamma_{ij}=0$$

Homogeneity condition (Equation (4)):

(4) $$\sum_{j}\gamma_{ij}=0$$

Symmetry condition (Equation (5)):

(5) $$\gamma_{ij}=\gamma_{ji}$$

The homogeneity condition requires budget shares to be invariant to proportional changes in all prices and total expenditure. In the AIDS model, this condition was imposed by constraining the sum of the price response parameters for each demand equation so that it equals zero (Equation (4)). This restriction ensures that the estimated demand relationships depend on relative rather than absolute prices. The symmetry condition was imposed by constraining the cross-price response parameters to be symmetric across commodity pairs (Equation (5)). This restriction reflected the symmetry of compensated substitution effects implied by consumer demand theory. Both homogeneity and symmetry restrictions were imposed during estimation to ensure theoretical consistency of the demand system. The AIDS demand system was estimated using Seemingly Unrelated Regression (SUR) to account for contemporaneous correlation across demand equations [25,26]. To satisfy the theoretical adding-up restriction and avoid singularity in the variance–covariance matrix, the ‘other food’ category was omitted from the system [24]. The parameters of the omitted ‘other’ category s were implicitly recovered by imposing homogeneity and symmetry constraints (Equations (4) and (5)) during the estimation process [27].

### 2.7. Elasticity Estimation

Elasticities were computed post-estimation from the estimated parameters of the Almost Ideal Demand System (AIDS) using mean budget shares computed for each estimation group, following Deaton and Muellbauer (1980) [11] and Green and Alston (1990) [28]. The resulting elasticity estimates characterize the magnitude and direction of consumer responses to changes in prices and total household food expenditure. Standard errors of the elasticity estimates were calculated using the delta method based on the variance–covariance matrix of the estimated AIDS parameters. Elasticity estimates and their standard errors were obtained post-estimation using nonlinear combinations of the estimated parameters. Elasticities were evaluated at the mean budget shares of each estimation group. Elasticities are derived as shown in Equations (6)–(8):

(6) $$\varepsilon_{ii}=-1+\;\frac{\gamma_{ii}}{w_{i}}-\;\beta_{i},$$

(7) $$\varepsilon_{ij}=\frac{\gamma_{ij}}{w_{i}}-\beta_{i},\;i\neq j,$$

(8) $$\eta_{i}=1+\frac{\beta_{i}}{w_{i}}.$$

The own-price elasticity $\varepsilon_{ii}$ measures the responsiveness of demand for a commodity i to changes in its own price, while the cross-price elasticity $\varepsilon_{ij}$ captures the substitution or complementarity effects between commodities i and j. The expenditure elasticity $\eta_{i}$ reflects the responsiveness of demand to changes in total household food expenditure. All elasticities are evaluated at the mean budget shares of each estimation group [29].

## 3. Results and Discussion

### 3.1. Expenditure Patterns

#### 3.1.1. Household Expenditure Patterns

Data on household expenditures derived from Susenas 2024 were analyzed to capture variations in consumption patterns across regions and socioeconomic groups. Table 1 shows that urban households had significantly higher total expenditure than rural households (IDR 1,737,427 ± 1,772,362 vs. IDR 1,162,944 ± 801,681 per capita per month). Rural households allocated a larger share of their expenditure to food than urban households (52.2% vs. 47.4%), whereas urban households allocated a larger share to non-food expenditure (52.6% vs. 47.8%). These differences were statistically significant for all expenditure indicators (all *p* < 0.001). Across expenditure quintiles, total expenditures increase markedly from IDR 553,520 in Quintile 1 to IDR 3,444,061 in Quintile 5, while the share allocated to food declined from 56.4% to 39.5% and the non-food share increased correspondingly. This pattern is consistent with Engel’s law, whereby the proportion of household expenditure allocated to food decreases as income increases, while expenditure on non-food goods and services becomes relatively more important [30,31].

The urban–rural differences may reflect differences in access to markets, goods, and services, with urban households potentially having greater opportunities for diversified consumption [32,33,34]. Similarly, the declining food expenditure share across expenditure quintiles is consistent with broader consumption diversification as household welfare increases [14,35]. The pattern also reflects the relatively lower income responsiveness of food expenditure compared with many non-food goods, as described by Engel’s law [36].

From a policy perspective, the relatively larger food expenditure share among lower-expenditure households highlights the importance of considering affordability and food expenditure structure when assessing the equity implications of food fortification [14]. Because these households devote a greater proportion of their resources to food, fortification of widely consumed staple commodities may offer substantial potential reach among nutritionally vulnerable populations [35]. However, expenditure patterns alone do not establish actual fortification coverage or nutritional benefit, which depend on commodity consumption, fortification compliance, and nutrient intake [36].

#### 3.1.2. Expenditure Shares on Mandatory Fortification Vehicles

The analysis of expenditure patterns on mandatory fortified food vehicles—wheat flour and its derivatives, palm cooking oil, and salt—reveals systematic differences in both absolute spending and budget shares across regions and expenditure groups (Table 2). At the national level, wheat flour and its processed products account for the largest expenditure among the fortification vehicles examined, averaging IDR 91,536 per capita per month and representing 6.1% of total food expenditure. Palm cooking oil accounts for 1.1%, while salt represents only 0.1%, reflecting its low unit cost despite widespread consumption. These differences indicate distinct economic roles of the three commodities within household food expenditure patterns [30,37].

Regional patterns also differ across vehicles. Urban households spend more on wheat flour and its derivatives in absolute terms than rural households (IDR 101,683 vs. IDR 77,073 per capita per month), whereas the expenditure share is higher in rural households (6.6% vs. 5.9%). Palm cooking oil shows similar absolute expenditure between urban and rural households, although its expenditure share is higher in rural areas (1.4% vs. 0.9%). Salt expenditure is minimal in both settings, with a similar expenditure share of 0.1%. These patterns may reflect differences in consumption structure and access to processed and convenience foods across regions [32,33,34].

Across expenditure quintiles, absolute spending on all three vehicles increases as household expenditure rises, while their budget shares generally decline. Wheat expenditure increases from IDR 44,894 per capita per month in Quintile 1 to IDR 161,992 in Quintile 5, while its share declines from 8.1% to 4.7%. Similarly, palm cooking oil expenditure increases from IDR 9624 to IDR 23,960, whereas its share declines from 1.7% to 0.7%. Salt expenditure also increases modestly, from IDR 1045 to IDR 2081, while its expenditure share remains low at 0.1–0.2%. This pattern is consistent with an Engel-type relationship in which the relative budget allocation to staple commodities declines as household welfare increases [21,38,39].

From a policy perspective, the larger budget shares allocated to these vehicles among households in the lower expenditure quintiles indicate a greater reliance on these commodities within their food expenditure structure [22]. However, expenditure shares alone cannot be used to infer the distribution of nutritional benefits from fortification, because nutritional exposure depends on actual consumption quantities, fortification status, and nutrient content [40,41]. Therefore, these findings primarily characterize the economic importance of the three fortification vehicles within household food budgets, while their consumption patterns and potential population reach are examined in the subsequent sections.

### 3.2. Consumption of Mandatory Fortification Vehicles

The results presented in Table 3 reveal clear variation in the consumption of mandatory fortification vehicles—wheat flour and its derivatives, palm cooking oil, and salt—across regions and expenditure quintiles in Indonesia. These differences indicate that the three commodities have distinct consumption patterns across population groups, which are relevant when considering their potential reach as fortification vehicles.

From a regional perspective, urban households exhibit higher per capita consumption of wheat flour and its derivatives (20.37 kg/cap/year) than rural households (18.08 kg/cap/year). This difference may reflect differences in food consumption structures and access to processed and convenience foods, including noodles, bread, and baked products [32,33,34]. In contrast, palm cooking oil consumption was almost identical between urban and rural households (9.53 vs. 9.52 kg/capita/year), indicating relatively consistent consumption across geographic settings. Salt consumption was higher among rural than urban households (1.28 vs. 0.95 kg/capita/year). These differences may reflect variations in dietary practices, food preparation, and the relative contribution of home-prepared foods across settings. However, these explanations should be interpreted as plausible contextual factors rather than causal relationships.

Across expenditure quintiles, wheat flour and its derivatives showed the largest socioeconomic gradient, increasing from 13.99 kg/cap/year in the lowest quintile to 25.47 kg/cap/year in the highest quintile. Palm cooking oil also increased across expenditure quintiles, from 7.19 kg/cap/year to 11.56 kg/cap/year, although the gradient was less pronounced than for wheat flour. In contrast, salt consumption remained relatively stable across all quintiles, ranging from 0.99 to 1.16 kg/cap/year across the expenditure distribution. The stronger increase in wheat consumption with household expenditure is consistent with broader patterns of dietary diversification, in which consumption of processed and higher-value foods tends to increase as household welfare improves [14,42]. Recent evidence from Indonesia similarly indicates structural changes in household food consumption and declining reliance on staple foods as expenditure increases [43].

These patterns have different implications for the potential reach of fortification vehicles. The relatively strong socioeconomic gradient in wheat flour consumption suggests greater potential exposure among higher-expenditure households, whereas the more consistent consumption of palm cooking oil across expenditure groups indicates a broader distribution of potential exposure. Salt shows the most stable consumption pattern across expenditure groups, suggesting broad potential reach across the expenditure distribution. Nevertheless, these findings describe consumption of the commodities irrespective of fortification status and therefore should not be interpreted as evidence of actual fortified-product coverage or nutritional benefit.

Overall, the consumption patterns demonstrate that the three mandatory fortification vehicles differ in their distribution across geographic and socioeconomic groups. These differences provide an important basis for interpreting the participation rates presented in the next section and the demand responsiveness estimates presented subsequently.

### 3.3. Consumption Participation Rates of Fortification Vehicles

**Figure 1:**
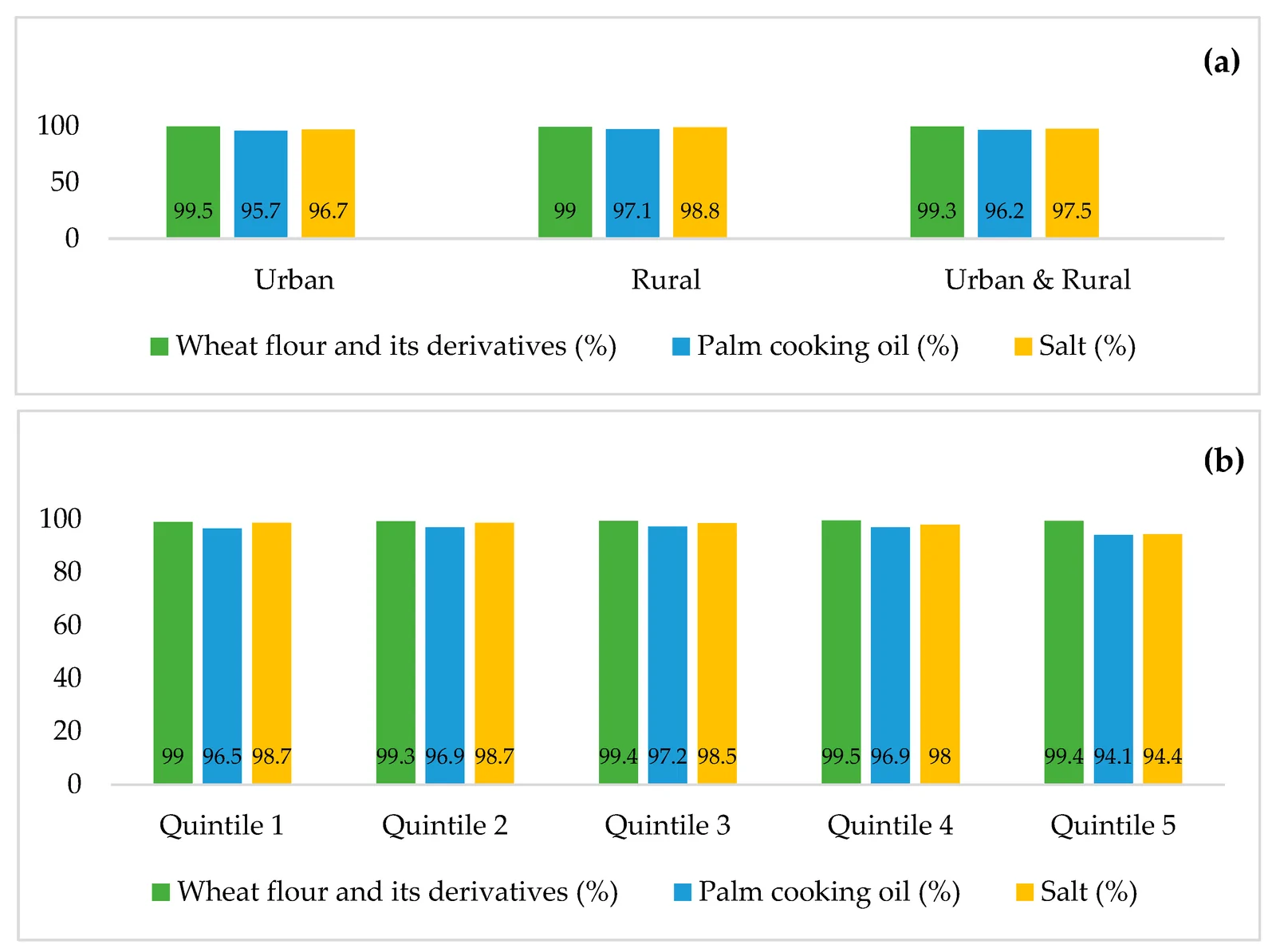
Household consumption participation rates of fortification vehicles (%): (**a**) by region and (**b**) by expenditure quintile.

Participation rates for wheat flour and its derivatives, palm cooking oil, and salt were very high across the population (Figure 1). At the national level, wheat flour and its derivatives had the highest participation rate (99.3%), followed by salt (97.5%) and palm cooking oil (96.2%). Participation rates also varied only modestly between urban and rural households, indicating that all three commodities were widely consumed across geographic settings.

Wheat flour and its derivatives showed near-universal participation, with rates of 99.5% in urban households and 99.0% in rural households. Participation remained similarly high across expenditure quintiles, ranging from 99.0% to 99.5%. These findings indicate that wheat-based products are widely present in household diets across Indonesia, geographic and socioeconomic groups, despite the differences in consumption quantities observed across expenditure quintiles.

Palm cooking oil also showed high participation, with a national average of 96.2%, and participation being slightly higher in rural (97.1%) than in urban (95.7%) households. Across expenditure quintiles, participation ranged from 94.1% to 97.2%, with the lowest rate observed in Quintile 5. This relatively limited variation indicates that palm cooking oil is widely present in household diets across expenditure groups, although its participation is somewhat lower among the highest expenditure households.

Salt showed similarly high participation rates, reaching 97.5% nationally, with higher participation in rural (98.8%) than urban households (96.7%). Participation rates remained high across expenditure quintiles, although it declined to 94.4% in Quintile 5. Overall, the relatively small variation across expenditure groups indicates that salt is widely present in household diets across the expenditure distribution.

Taken together, the high participation rates indicate that all three commodities have substantial potential reach as food fortification vehicles. However, participation rates reported in this study reflect consumption of the commodity regardless of fortification status. Therefore, these estimates should not be interpreted as actual coverage of fortified products. Actual fortification coverage depends on the availability and consumption of fortified products, compliance with fortification standards, and the effectiveness of regulatory monitoring and quality control [44,45]. Similarly, high participation does not imply equivalent nutritional exposure, because nutrient exposure also depends on the quantity consumed, fortification level, and intra-household distribution [42,44]. This distinction is supported by recent global modeling evidence showing that the nutritional impact of large-scale food fortification depends not only on the existence of fortification programmes but also on programme compliance and coverage [46]. Improving compliance with existing standards was estimated to produce substantial additional reductions in micronutrient inadequacies, indicating that high potential reach alone is insufficient to ensure nutritional impact.

From a policy perspective, the findings suggest that the three mandatory fortification vehicles have broad potential reach, but their distribution should be considered alongside consumption quantities and demand responsiveness. Wheat flour and its derivatives show the highest participation but also a stronger socioeconomic gradient in consumption quantity, whereas palm cooking oil and salt show relatively stable participation across expenditure groups. These complementary characteristics support consideration of multiple fortification vehicles rather than reliance on participation rates alone when assessing population reach and equity.

### 3.4. Demand Elasticity and Consumption Modeling of Mandatory Fortification Vehicles

#### 3.4.1. Wheat Flour and Derivatives

The estimated demand elasticities for wheat flour and its derived products are presented in Table 4. At the national level, the own-price elasticity is estimated at −0.848, indicating that a 1% increase in the price of wheat flour and its derivatives was associated with an approximately 0.85% reduction in demand. Thus, demand was price inelastic at the national level, although the magnitude close to unit elasticity indicates that consumption was still responsive to price changes.

Regional estimates show lower absolute price elasticity in urban households (−0.490) than in rural areas (−0.606), indicating greater price responsiveness in rural areas. This difference indicates greater price responsiveness among rural households, although the underlying mechanisms were not directly examined in this study.

Substantial variation was also observed across expenditure quintiles. The lowest-expenditure quintile (quintile 1) had an own-price elasticity (−1.043), indicating a slightly elastic demand, whereas the higher-expenditure quintiles showed inelastic demand, with absolute elasticities below one. This pattern indicates greater price responsiveness among households in the lowest expenditure quintile. However, because formal statistical tests comparing elasticity estimates across expenditure groups were not conducted, differences in elasticity magnitudes should be interpreted as descriptive patterns rather than statistically confirmed between-group differences.

Cross-price elasticities were generally small in magnitude, indicating limited associations between wheat flour demand and the prices of other food commodities. At the national level, the negative cross-price elasticity with rice (−0.190) and positive elasticity with fish (0.287) indicate complementary and substitution relationships, respectively. However, given the generally small magnitudes of the cross-price estimates, these relationships appear to play a relatively limited role in determining wheat flour demand compared with its own price and household expenditure.

Expenditure elasticities were positive across all geographic and expenditure groups, confirming that wheat flour and its derivatives behaved as normal goods. The national expenditure elasticity is estimated at 0.988, indicating that a 1% increase in household expenditure was associated with an approximately 0.99% increase in wheat consumption. Responsiveness was slightly higher in rural areas (1.013) compared with urban areas (0.951). Across quintiles, the elasticity was highest in Quintile 1 and declined to 0.855 in Quintile 5, indicating greater expenditure responsiveness among lower-expenditure households.

From a fortification policy perspective, these findings indicate that wheat flour and its derivatives combine very high participation with relatively stable national demand, but the distribution of consumption and demand responsiveness has important equity implications. Lower-expenditure households showed greater sensitivity to price changes and higher expenditure responsiveness, while wheat consumption quantities were also substantially lower among these households. Therefore, maintaining affordability and minimizing unintended price barriers are important considerations when assessing the potential reach of wheat flour fortification among lower-expenditure households [35]. At the same time, elasticity estimates should be interpreted as evidence of demand responsiveness and affordability rather than as direct evidence of fortification effectiveness, because nutritional impact also depends on the quantity consumed, fortification status, compliance, and nutrient delivery [44].

Overall, while wheat flour is a widely consumed fortification vehicle, the socioeconomic gradient in consumption and greater price sensitivity among lower-expenditure households suggest that consumption-based potential reach may not translate into equivalent exposure across population groups. This reinforces the value of considering wheat flour alongside other fortification vehicles with different consumption distributions.

#### 3.4.2. Palm Cooking Oil

The estimated demand elasticities for palm cooking oil are presented in Table 5. At the national level, the own-price elasticity was −0.499, indicating that a 1% increase in the price of palm cooking oil was associated with an approximately 0.50% reduction in demand. Thus, demand for palm cooking oil was price-inelastic, suggesting that household consumption was relatively less responsive to changes in its own price than to proportional changes in price. The finding is consistent with previous evidence characterizing palm cooking oil as a staple and normal good in Indonesia [47].

Regional estimates showed an own-price elasticity of −0.559 in urban households and −0.389 in rural households. Both estimates indicate price-inelastic demand, although urban households showed greater price responsiveness in absolute terms. These differences should be interpreted cautiously, as the mechanisms underlying the regional variation were not directly examined in this study.

Across expenditure quintiles, own-price elasticities were also generally inelastic, ranging from −0.226 in Quintile 4 to −0.740 in Quintile 3. The highest absolute elasticity was observed in Quintile 3 (−0.740), followed by Quintile 1 (−0.723), whereas Quintile 4 showed a relatively small and statistically non-significant estimate (−0.226). This pattern indicates heterogeneity in price responsiveness across expenditure groups rather than a clear monotonic relationship between household expenditure and price sensitivity. Because formal statistical tests comparing elasticity estimates across expenditure groups were not conducted, these differences should be interpreted as descriptive patterns rather than statistically confirmed differences between groups.

The cross-price elasticity estimates reveal generally small and mixed relationships between palm cooking oil and other food commodities. At the national level, the positive cross-price elasticity with rice (0.218) suggests a substitution relationship, whereas negative elasticities with respect to wheat flour and derived products (−0.108), eggs and milk (−0.201), fish (−0.118), and meat (−0.082) indicate complementary relationships. However, the relatively small magnitude of, and variation in, these estimates across regions and expenditure groups suggest that changes in the prices of other food commodities were not consistently associated with large changes in palm cooking oil demand. These relationships should therefore be interpreted as conditional associations within the estimated demand system rather than as evidence of strong substitution or complementarity.

Expenditure elasticities were positive across all geographic and expenditure groups, indicating that palm cooking oil behaved as a normal good. The national expenditure elasticity was 0.619, meaning that a 1% increase in household food expenditure was associated with an approximately 0.62% increase in palm cooking oil consumption. Regional estimates were somewhat higher in rural households (0.431) than in urban households (0.364). Across expenditure quintiles, expenditure responsiveness varied considerably: elasticity was highest in Quintile 1 (1.723), followed by Quintile 4 (1.501), Quintile 2 (1.331), Quintile 3 (1.162), and Quintile 5 (0.734). Thus, expenditure responsiveness was greater than proportional in Quintiles 1–4 but less than proportional in Quintile 5. The non-monotonic pattern suggests that the relationship between household expenditure and palm cooking oil consumption is heterogeneous across expenditure groups rather than following a simple linear gradient.

From a fortification policy perspective, the combination of high participation and nationally price-inelastic demand suggests that palm cooking oil has substantial potential reach and that aggregate consumption may be relatively resilient to moderate price changes. However, the heterogeneity observed across expenditure groups indicates that this potential reach should be considered alongside affordability and consumption responsiveness. In particular, the relatively higher price responsiveness observed in Quintiles 1 and 3 suggests that price changes may affect consumption more strongly in some lower- and middle-expenditure groups. Evidence from Indonesia also indicates that cooking oil is a staple commodity and that price conditions can affect consumer welfare, supporting the importance of maintaining affordability when designing policies involving cooking oil [47,48].

Overall, palm cooking oil represents a widely consumed fortification vehicle with relatively inelastic demand at the national level. Nevertheless, differences in price and expenditure responsiveness across population groups indicate that consumption stability should not be assumed to be uniform across households. Moreover, elasticity estimates describe demand responsiveness and affordability rather than actual fortification effectiveness, which also depends on the consumption of fortified products, compliance with fortification standards, and nutrient delivery [35,49].

#### 3.4.3. Salt

The estimated demand elasticities for salt are presented in Table 6. At the national level, the own-price elasticity of salt is estimated at −0.844, indicating that a 1% increase in price was associated with an approximately 0.84% reduction in demand. Thus, salt demand was price-inelastic, although its responsiveness to price changes was greater than that observed for palm cooking oil. Regional estimates were also price-inelastic, with elasticities of −0.699 in urban households and −0.785 in rural households.

Across expenditure quintiles, own-price elasticity ranges from −0.786 to −0.969, with all estimates statistically significant and remaining below unity in absolute value. The highest absolute elasticity was observed in Quintile 4 (−0.969), followed by Quintile 5 (−0.923). Because formal statistical tests comparing elasticity estimates across expenditure groups were not conducted, differences in magnitude should be interpreted as descriptive patterns rather than statistically confirmed between-group differences. Overall, these results indicate relatively stable salt demand across geographic and expenditure groups in response to price changes.

Cross-price elasticities between salt and other food commodities were generally heterogeneous. Although the national elasticity with respect to rice was positive and relatively large (1.000), relationships with other commodities varied across geographic and expenditure groups. Given this heterogeneity, cross-price effects are interpreted cautiously and are not the primary basis for policy interpretation.

Expenditure elasticity was positive across all geographic and expenditure groups, indicating that salt behaved as a normal good. The national expenditure elasticity was 0.571, with estimates of 0.187 in urban households and 0.487 in rural households. Across expenditure quintiles, expenditure elasticity ranged from 0.577 in Quintile 1 to 1.054 in Quintile 4, with an estimate of 1.013 in Quintile 5. This pattern indicates that the relationship between household expenditure and salt consumption was not monotonic across expenditure groups.

From a fortification policy perspective, salt combines high participation with relatively stable and price-inelastic demand, indicating substantial potential reach as a fortification vehicle. However, the potential reach observed in this study should not be interpreted as actual coverage of fortified salt or as evidence of nutritional impact. Effective fortification requires adequate availability of fortified products, compliance with fortification standards, quality assurance, and regulatory monitoring. These implementation factors are particularly important for salt because its widespread consumption creates an opportunity for population-level delivery, but consumption alone does not ensure that households receive adequately fortified products [44,45].

Taken together, the findings indicate that the three mandatory fortification vehicles have distinct consumption and demand characteristics with important implications for potential population reach and equity. Wheat flour and its derivatives had the highest participation rate and showed substantial socioeconomic variation in consumption, together with greater price responsiveness among lower-expenditure households. Palm cooking oil showed high and relatively stable participation, although demand responsiveness varied across expenditure groups. Salt showed high participation and relatively inelastic demand across geographic and expenditure groups, while its expenditure responsiveness varied across the expenditure distribution. These differences indicate that no single indicator—whether participation, consumption quantity, or demand elasticity—is sufficient to characterize the population-level potential of a fortification vehicle. Rather, vehicle selection and policy implementation should consider the distribution of consumption, affordability, and demand responsiveness across population groups. Importantly, the high potential reach identified in this study should not be equated with actual fortification coverage or nutritional impact, which also depend on fortification standards, programme coverage, compliance, and effective regulatory monitoring [35,44,46].

These findings should also be considered alongside implementation feasibility. Although this study did not estimate programme costs or directly assess consumer acceptance and sensory characteristics, these factors are relevant to the feasibility and sustainability of food fortification programmes. The selection and implementation of fortification vehicles should consider consumption profiles, affordability, programme costs, technological feasibility, and potential sensory or physical effects [35]. Programme costs may arise from premix, industry, and government activities, while effective quality assurance and regulatory monitoring are needed to ensure compliance with fortification standards [45,46]. Thus, high potential reach based on commodity consumption should be complemented by attention to implementation feasibility, product quality, and compliance when assessing the potential effectiveness of fortification programmes.

## 4. Conclusions

This study provides a nationally representative analysis of household consumption patterns, participation rates, and demand elasticities for Indonesia’s mandatory food fortification vehicles using Susenas 2024. Wheat flour and its derivatives, palm cooking oil, and salt were widely consumed, with participation rates exceeding 95% nationally. However, their consumption distributions and demand responses differed across geographic and expenditure groups. Wheat flour and its derivatives showed the highest participation rate and the largest socioeconomic gradient in consumption, while palm cooking oil showed relatively stable participation across expenditure groups. Salt also showed high participation and relatively stable consumption quantities across expenditure groups. Demand was generally price-inelastic at the national level, although the magnitude of price and expenditure responsiveness varied across fortification vehicles, expenditure groups, and urban–rural areas.

These findings indicate that the potential reach of a fortification vehicle cannot be assessed from participation rates alone. Consumption quantity, affordability, and demand responsiveness provide complementary information for understanding how fortification vehicles may reach different population groups. In particular, the greater price responsiveness observed for wheat flour among lower-expenditure households highlights the importance of maintaining affordability, while the heterogeneous elasticity patterns for palm cooking oil and salt indicate that socioeconomic differences should be considered when assessing potential population reach. Importantly, the high commodity participation observed in this study should not be interpreted as actual coverage of fortified products or as evidence of nutritional impact, which also depend on fortification standards, product availability, compliance, and effective monitoring.

From a policy perspective, the findings support a complementary, multi-vehicle approach to fortification rather than reliance on a single vehicle. Existing fortification programmes should continue to prioritize widely consumed vehicles while considering differences in consumption distribution and affordability across population groups. For wheat flour and palm cooking oil, affordability and potential price-related barriers warrant attention, particularly among groups showing greater demand responsiveness. For salt, maintaining high-quality iodization and strengthening regulatory monitoring and compliance remain important to translate its high potential reach into effective fortification coverage. More broadly, decisions to maintain, strengthen, or expand fortification vehicles should consider not only population consumption but also implementation feasibility, fortification costs, consumer acceptability, sensory characteristics, and the capacity of quality assurance and monitoring systems.

## Figures and Tables

**Table 1:** Household expenditure per capita per month by region and expenditure quintile.

Household Characteristics	Category	Food Expenditure (IDR/Cap/Month)	Food Share (%)	Non-Food Expenditure (IDR/Cap/Month)	Non-Food Share (%)	Total Expenditures (IDR/Cap/Month)
Region	Urban	718,022 ± 463,805	47.4 ± 11.2	1,019,405 ± 1,421,020	52.6 ± 11.2	1,737,427 ± 1,772,362
	Rural	570,785 ± 301,306	52.2 ± 10.8	592,159 ± 595,744	47.8 ± 10.8	1,162,944 ± 801,681
	Urban and Rural	657,313 ± 411,223	49.4 ± 11.3	843,243 ± 1,173,570	50.6 ± 11.3	1,500,556 ± 1,480,210
	*p*-value	<0.001	<0.001	<0.001	<0.001	<0.001
Expenditure quintile	Quintile 1	310,613 ± 71,788	56.4 ± 9.0	242,907 ± 71,825	43.6 ± 9.0	553,520 ± 103,300
	Quintile 2	438,015 ± 79,462	53.0 ± 8.8	389,159 ± 83,371	47.0 ± 8.8	827,174 ± 73,338
	Quintile 3	565,724 ± 109,929	50.6 ± 9.1	552,846 ± 117,124	49.4 ± 9.1	1,118,570 ± 97,907
	Quintile 4	736,637 ± 164,099	47.4 ± 9.7	822,830 ± 187,168	52.6 ± 9.7	1,559,467 ± 172,854
	Quintile 5	1,235,582 ± 526,947	39.5 ± 11.7	2,208,479 ± 2,076,121	60.5 ± 11.7	3,444,061 ± 2,371,224
	*p*-value	<0.001	<0.001	<0.001	<0.001	<0.001

Note: Values are presented as mean ± standard deviation (SD). *p*-values were obtained using the Complex Samples General Linear Model, accounting for the survey sampling design (strata, primary sampling units, and sampling weights).

**Table 2:** Expenditure on mandatory fortification vehicles per capita per month.

Household Characteristics	Category	Wheat Flour and Derivatives (Rp/Capita/Month)	Share of Wheat Flour Expenditure (%)	Palm Cooking Oil (Rp/Capita/Month)	Share of Palm Cooking Oil Expenditure (%)	Salt (Rp/Capita/Month)	Share of Salt Expenditure (%)	Total
Region	Urban	101,683 ± 74,818	5.9	15,815 ± 11,048	0.9	1293 ± 2890	0.1	118,790 ± 78,777
	Rural	77,073 ± 54,488	6.6	15,906 ± 11,679	1.4	1713 ± 1982	0.1	94,692 ± 58,877
	Urban and Rural	91,536 ± 68,269	6.1	15,853 ± 11,312	1.1	1466 ± 2563	0.1	108,854 ± 72,229
Expenditure Quintile	Quintile 1	44,894 ± 23,664	8.1	9624 ± 5585	1.7	1045 ± 897	0.2	55,562 ± 24,151
	Quintile 2	64,963 ± 31,134	7.9	12,626 ± 7102	1.5	1240 ± 1064	0.1	78,828 ± 31,216
	Quintile 3	82,167 ± 39,497	7.3	15,081 ± 8058	1.3	1390 ± 1623	0.1	98,637 ± 39,544
	Quintile 4	103,664 ± 50,596	6.6	17,974 ± 10,189	1.2	1575 ± 3075	0.1	123,213 ± 50,983
	Quintile 5	161,992 ± 97,844	4.7	23,960 ± 16,414	0.7	2081 ± 4266	0.1	188,032 ± 100,216

**Table 3:** Consumption of mandatory fortification food vehicles (kg/cap/year).

Household Characteristics	Category	Wheat Flour and Its Derivatives (kg/Cap/Year)	Palm Cooking Oil (kg/Cap/Year)	Salt (kg/Cap/Year)
Region	Urban	20.37 ± 12.66	9.53 ± 5.52	0.95 ± 0.79
	Rural	18.08 ± 12.05	9.52 ± 5.41	1.28 ± 0.94
	Urban and Rural	19.43 ± 12.46	9.53 ± 5.48	1.09 ± 0.87
Expenditure Quintile	Quintile 1	13.99 ± 8.73	7.19 ± 3.82	0.99 ± 0.76
	Quintile 2	17.22 ± 10.13	8.65 ± 4.41	1.07 ± 0.82
	Quintile 3	19.04 ± 11.14	9.66 ± 4.95	1.11 ± 0.86
	Quintile 4	21.41 ± 12.55	10.59 ± 5.73	1.16 ± 0.93
	Quintile 5	25.47 ± 15.53	11.56 ± 6.86	1.11 ± 0.97

**Table 4:** Own-price, cross-price, and expenditure elasticities of wheat flour and derived products.

Elasticity Type	Commodity/Group	National	Urban	Rural	Quintile 1	Quintile 2	Quintile 3	Quintile 4	Quintile 5
Own-price elasticity	Wheat flour and derived products	−0.848 *** (0.057)	−0.490 *** (0.076)	−0.606 *** (0.097)	−1.043 *** (0.112)	−0.591 *** (0.105)	−0.736 *** (0.125)	−0.633 *** (0.106)	−0.733 *** (0.103)
Cross-price elasticity	Rice	−0.190 *** (0.056)	−0.219 *** (0.054)	−0.057 (0.066)	0.049 (0.097)	−0.175 * (0.093)	−0.165 (0.108)	−0.117 (0.091)	−0.192 ** (0.090)
	Other staple foods	0.009 (0.017)	−0.019 (0.014)	−0.061 ** (0.030)	−0.007 (0.031)	−0.033 (0.034)	0.045 (0.037)	−0.009 (0.034)	−0.017 (0.042)
	Fish	0.287 *** (0.030)	0.068 * (0.035)	0.042 (0.046)	0.126 ** (0.057)	0.022 (0.058)	0.204 *** (0.062)	−0.049 (0.060)	0.055 (0.064)
	Meat	−0.077 *** (0.025)	−0.132 *** (0.031)	−0.182 *** (0.039)	−0.113 * (0.059)	−0.107 * (0.055)	−0.018 (0.065)	0.041 (0.054)	0.122 ** (0.058)
	Eggs and milk	−0.077 *** (0.026)	−0.125 *** (0.032)	−0.073 * (0.037)	−0.100 * (0.058)	−0.050 (0.054)	−0.116 * (0.062)	−0.059 (0.057)	−0.086 * (0.050)
	Vegetables and fruits	−0.013 (0.032)	−0.001 (0.040)	−0.052 (0.047)	0.048 (0.066)	−0.009 (0.065)	−0.087 (0.071)	−0.113 * (0.066)	−0.055 (0.074)
	Palm cooking oil	−0.021 * (0.011)	−0.038 *** (0.014)	0.004 (0.016)	−0.027 (0.028)	0.028 (0.027)	−0.052 ** (0.027)	−0.049 ** (0.019)	0.000 (0.018)
	Other oils and fats	0.002 (0.006)	0.006 (0.007)	0.002 (0.009)	−0.023 (0.015)	−0.029 ** (0.013)	0.003 (0.014)	0.008 (0.012)	0.006 (0.009)
	Legumes	−0.063 *** (0.014)	−0.012 (0.018)	−0.025 (0.020)	−0.051 (0.036)	−0.032 (0.032)	−0.058 * (0.031)	0.036 (0.027)	−0.001 (0.021)
	Salt	0.000 (0.002)	0.001 (0.002)	−0.002 (0.003)	−0.012 ** (0.006)	0.002 (0.005)	0.001 (0.005)	0.006 (0.004)	0.010 *** (0.003)
Expenditure elasticity	Wheat flour and derived products	0.988 *** (0.007)	0.951 *** (0.010)	1.013 *** (0.010)	1.203 *** (0.055)	0.969 *** (0.048)	0.975 *** (0.047)	0.921 *** (0.042)	0.855 *** (0.021)

Notes: Standard errors are reported in parentheses. * *p* < 0.10, ** *p* < 0.05, *** *p* < 0.01.

**Table 5:** Own-price, cross-price, and expenditure elasticities of palm cooking oil.

Elasticity Type	Commodity/Group	National	Urban	Rural	Quintile 1	Quintile 2	Quintile 3	Quintile 4	Quintile 5
Own-price elasticity	Palm cooking oil	−0.499 *** (0.069)	−0.559 *** (0.107)	−0.389 *** (0.097)	−0.723 *** (0.136)	−0.488 *** (0.171)	−0.740 *** (0.178)	−0.226 (0.189)	−0.330 ** (0.151)
Cross-price elasticity	Rice	0.218 ** (0.086)	−0.061 (0.129)	−0.255 ** (0.112)	−0.186 (0.189)	−0.028 (0.197)	0.404 ** (0.197)	0.105 (0.185)	0.485 ** (0.193)
	Wheat flour and derived products	−0.108 * (0.057)	−0.228 *** (0.078)	0.031 (0.078)	−0.066 (0.112)	0.120 (0.133)	−0.292 ** (0.141)	−0.330 *** (0.113)	−0.064 (0.123)
	Other staple foods	−0.037 (0.028)	0.030 (0.045)	−0.056 (0.046)	−0.105 (0.066)	−0.086 (0.072)	−0.183 *** (0.066)	0.021 (0.061)	−0.087 (0.071)
	Fish	−0.118 *** (0.039)	0.018 (0.057)	0.179 *** (0.061)	−0.096 (0.071)	−0.216 ** (0.089)	−0.115 (0.090)	−0.101 (0.086)	0.091 (0.111)
	Meat	−0.082 * (0.042)	0.044 (0.070)	0.083 (0.064)	0.292 *** (0.110)	−0.437 *** (0.128)	−0.248 ** (0.120)	−0.440 *** (0.107)	−0.490 *** (0.091)
	Eggs and milk	−0.201 *** (0.041)	−0.012 (0.060)	−0.153 *** (0.055)	−0.210 * (0.117)	−0.143 (0.119)	−0.160 (0.105)	−0.262 *** (0.087)	−0.205 *** (0.079)
	Vegetables and fruits	−0.053 (0.048)	−0.187 *** (0.073)	−0.012 (0.062)	0.037 (0.106)	0.074 (0.122)	0.243 ** (0.111)	0.102 (0.107)	−0.128 (0.127)
	Other oils and fats	−0.014 (0.025)	0.071 * (0.040)	−0.069 ** (0.034)	0.051 (0.056)	0.140 ** (0.064)	0.037 (0.063)	−0.020 (0.064)	−0.114 ** (0.051)
	Legumes	−0.028 (0.041)	−0.029 (0.061)	−0.235 *** (0.057)	−0.078 (0.084)	0.005 (0.100)	0.058 (0.102)	0.073 (0.090)	−0.133 (0.093)
	Salt	−0.022 ** (0.009)	0.004 (0.014)	−0.033 *** (0.013)	−0.051 *** (0.020)	0.005 (0.021)	−0.027 (0.021)	0.011 (0.022)	0.006 (0.019)
Expenditure elasticity	Palm cooking oil	0.619 *** (0.034)	0.364 *** (0.052)	0.431 *** (0.044)	1.723 *** (0.173)	1.331 *** (0.182)	1.162 *** (0.151)	1.501 *** (0.148)	0.734 *** (0.092)

Notes: Standard errors are reported in parentheses. * *p* < 0.10, ** *p* < 0.05, *** *p* < 0.01.

**Table 6:** Own-price, cross-price, and expenditure elasticities of salt.

Elasticity Type	Commodity/Group	National	Urban	Rural	Quintile 1	Quintile 2	Quintile 3	Quintile 4	Quintile 5
Own-price elasticity	Salt	−0.844 *** (0.027)	−0.699 *** (0.046)	−0.785 *** (0.036)	−0.805 *** (0.060)	−0.786 *** (0.059)	−0.880 *** (0.062)	−0.969 *** (0.056)	−0.923 *** (0.056)
Cross-price elasticity	Rice	1.000 *** (0.150)	0.390 * (0.229)	−0.096 (0.160)	−0.005 (0.292)	0.264 (0.269)	0.404 (0.288)	−0.261 (0.299)	1.151 *** (0.303)
	Wheat flour and derived products	−0.030 (0.099)	−0.155 (0.138)	−0.027 (0.111)	0.068 (0.172)	0.011 (0.173)	−0.027 (0.190)	0.048 (0.186)	−0.013 (0.202)
	Other staple foods	−0.199 *** (0.050)	−0.059 (0.083)	0.083 (0.066)	0.011 (0.108)	−0.070 (0.106)	−0.163 (0.106)	0.033 (0.107)	−0.061 (0.117)
	Fish	−0.476 *** (0.067)	−0.155 (0.101)	−0.003 (0.086)	−0.382 *** (0.128)	−0.252 ** (0.117)	−0.157 (0.124)	−0.007 (0.138)	−0.084 (0.173)
	Meat	−0.030 (0.077)	0.075 (0.127)	0.226 ** (0.093)	0.198 (0.171)	−0.058 (0.176)	−0.194 (0.177)	−0.267 (0.181)	−0.706 *** (0.161)
	Eggs and milk	−0.215 *** (0.069)	0.103 (0.108)	−0.045 (0.078)	−0.029 (0.188)	−0.011 (0.158)	−0.271 * (0.145)	−0.103 (0.142)	−0.171 (0.127)
	Vegetables and fruits	−0.153 * (0.079)	−0.508 *** (0.132)	−0.059 (0.089)	0.244 (0.166)	−0.152 (0.160)	0.134 (0.155)	0.036 (0.171)	−0.378 * (0.201)
	Palm cooking oil	−0.278 *** (0.095)	−0.050 (0.162)	−0.388 *** (0.120)	−0.365 ** (0.186)	0.099 (0.208)	−0.264 (0.219)	0.180 (0.236)	0.031 (0.208)
	Other oils and fats	−0.038 (0.046)	−0.067 (0.079)	0.015 (0.054)	−0.004 (0.109)	−0.124 (0.106)	0.054 (0.102)	0.070 (0.109)	0.060 (0.085)
	Legumes	0.271 *** (0.071)	0.134 (0.113)	0.087 (0.086)	0.078 (0.139)	0.082 (0.144)	0.367 ** (0.154)	0.239 (0.147)	0.093 (0.149)
Expenditure elasticity	Salt	0.571 *** (0.025)	0.187 *** (0.057)	0.487 *** (0.037)	0.577 *** (0.103)	0.845 *** (0.091)	0.804 *** (0.083)	1.054 *** (0.083)	1.013 *** (0.079)

Notes: Standard errors are reported in parentheses. * *p* < 0.10, ** *p* < 0.05, *** *p* < 0.01.

## Data Availability

The data used in this study were obtained from the 2024 National Socioeconomic Survey (Susenas) conducted by Statistics Indonesia (Badan Pusat Statistik, BPS) through the SILASTIK (Statistical Information System) service. The dataset is a third-party dataset and is subject to BPS data access and confidentiality regulations. The data are not publicly available from the authors but can be obtained from BPS through the SILASTIK service upon request and subject to the applicable data access requirements.

## Supplementary Materials

The following supporting information can be downloaded at: https://www.mdpi.com/article/10.3390/foods15183253/s1, Table S1: Susenas 2024 questionnaire modules used in the present study; Table S2: Household consumption participation rates of fortification vehicles (%).
