# Supplementary material for: Mandatory Food Fortification Vehicles in Indonesia: Consumption Patterns and Demand Elasticities from Susenas 2024
Source: Foods. 2026 Sep 15;15(18):3253. doi: 10.3390/foods15183253 (PMC13605840; doi:10.3390/foods15183253)
Supplement: Supplementary file 1 [file foods-15-03253-s001.zip › foods-4488818-supplementary.pdf]

Supplementary Data

Mandatory Food Fortification Vehicles in Indonesia: Consumption Patterns and Demand Elasticities from Susenas 2024

Drajat Martianto[1], Azis Boing Sitanggang[2,3,*], Jonathan Louis Gorstein[4], Nina Sardjunani[5], Roza Kartika[5], Elsa Fajriah[5] and Milatul Mustaqimah[5]

1 Division of Food and Nutrition Policy, Faculty of Medicine and Nutrition, IPB University, Bogor 16680, Indonesia
2 Division of Food Science and Technology, Faculty of Engineering and Technology, IPB University, Bogor 16680, Indonesia
3 Graduate School of Agricultural Science, Tohoku University, Sendai 980-8572, Miyagi, Japan
4 Gates Foundation, Seattle, WA 98109, USA
5 Indonesian Nutrition Foundation for Food Fortification (KFI), Komplek Bappenas A1, Jl. Siaga Raya, Pejaten Barat, Jakarta Selatan 12510, Indonesia
* Correspondence: boing.lipan@apps.ipb.ac.id

Table S1. Susenas 2024 questionnaire modules used in the present study.

No	Instrument	Information used
1	KOR/VSEN24.K	Household demographic and socioeconomic characteristics
2	KP/VSEN24.KP	Quantity and expenditure of food commodities

Table S2. Household consumption participation rates of fortification vehicles (%).

Household Characteristics	Category	Wheat flour and its derivatives (%)	Palm cooking oil (%)	Salt (%)
Region	Urban	99.5	95.7	96.7
	Rural	99.0	97.1	98.8
	Urban and Rural	99.3	96.2	97.5
Expenditure Quintile	Quintile 1	99.0	96.5	98.7
	Quintile 2	99.3	96.9	98.7
	Quintile 3	99.4	97.2	98.5
	Quintile 4	99.5	96.9	98.0
	Quintile 5	99.4	94.1	94.4
